# Utility of human papillomavirus L1 capsid protein and HPV test as prognostic markers for cervical intraepithelial neoplasia 2+ in women with persistent ASCUS /LSIL cervical cytology

**DOI:** 10.7150/ijms.31163

**Published:** 2019-08-06

**Authors:** Eun Young Ki, Jong Sup Park, Ahwon Lee, Tae Jung Kim, Hyun Tak Jin, Yong Bok Seo, Yuki Gen, Mi Young Park, Sung Jong Lee

**Affiliations:** 1 Department of Obstetrics and Gynecology, Daejeon St. Mary's Hospital, College of Medicine, The Catholic University of Korea; 2 Department of Obstetrics and Gynecology, Seoul St. Mary's Hospital, College of Medicine, The Catholic University of Korea, Seoul, Korea; 3 Department of Hospital Pathology, The Catholic University of Korea, College of Medicine, Seoul, Korea; 4 Research Institute, SL BIGEN, Inc., Korea Bio Park, Seongnam, Korea; 5 Department of Obstetrics and Gynecology, St. Vincent's Hospital, College of Medicine, The Catholic University of Korea, Suwon, Korea; 6 Cancer Research Institute, College of Medicine, The Catholic University of Korea, Seoul, Korea

**Keywords:** Cervical Intraepithelial Neoplasia, Human papillomavirus 16, Human papillomavirus 18, Low grade squamous intraepithelial lesion, Atypical squamous cells of unknown significance

## Abstract

**Objective:** Efficient and highly predictive biomarkers reflecting the prognosis of persistent atypical squamous cells of unknown significance(ASCUS) and low grade squamous intraepithelial lesion(LSIL)s are unavailable and need to be developed urgently. We aimed to develop a predictive model for diagnosis of cervical intraepithelial neoplasia(CIN)2+ by analyzing the immunocytochemical expression of the HPV L1 capsid protein in patients with persistent ASCUS and LSIL with a high risk of HPV infection.

**Methods**: Cervical cytology samples comprising (70 ASCUS and 215 LSIL Pap smears) were analyzed. Immunocytochemical identification of the HPV L1 capsid protein in cervical cytology samples was performed. Expression levels of HPV L1 capsid protein in cervical cytology samples were measured, and the correlation between HPV L1 expression and cervical pathologic diagnosis was evaluated. The risk for CIN2+ was calculated using the results of immunocytochemistry and the HPV DNA test.

**Results**: Negative results for HPV L1 immunochemistry test were more frequently observed in CIN2+, and expression of the HPV L1 capsid protein was higher in CIN1 or cervicitis (Fisher's exact test, *p*<0.05). Diagnosis rates for CIN2+ were highest for the combination of HPV L1 capsid protein immunocytochemistry, cytology and HPV test when compared with other combinations (Akaike information criterion (AIC): 191.7, Schwarz criterion(SC): 206.3, p<0.001).

**Conclusion:** Absence of HPV L1 capsid expression and presence of HPV type 16 or 18 infection are reliable predictors of progression to CIN2+ in patients showing persistent ASCUS and LSIL.

## Introduction

Human papillomavirus (HPV) is an important cause of cervical cancer and cervical intraepithelial neoplasia (CIN), and is associated with 99% of cervical cancer^1^. In an effort to reduce the incidence of cervical cancer, cervical cytology has been adopted as a screening method for cervical disease. Minor abnormal cytology, such as atypical squamous cells of unknown significance (ASCUS) or low grade squamous intraepithelial lesions (LSIL), are the possible manifestations of transient HPV infection. The American Society for Colposcopy and Cervical Pathology (ASCCP) recommends observation instead of intervention due to the high likelihood of spontaneous regression. However, there are a few contradictions between ASCCP guidelines and clinical practice in the management of ASCUS and LSIL^2^.

Despite the high probability of spontaneous regression of ASCUS and LSIL, persistent minor cervical abnormalities are occasionally observed in women with HPV infection^3^. Most patients are concerned about the possibility of disease progression to a high grade cervical lesion^4^. Up to 6% of ASCUS and 30% of LSIL presented with CIN 2+( including CIN2, CIN3, CIS, AIS, and cervical cancer)^5^, ^6^. Identification of individuals with ASCUS or LSIL at high risk for developing CIN 2+ is thus clinically important. Recently, immunohistochemistry of p16 and Ki-67 was utilized to provide a more accurate diagnosis of high grade CIN. In the ASCUS-LSIL triage study (ALTS) trial, the HPV test and colposcopy were used in combination to enhance the detection rate of high grade cervical disease^7^. However, this approach did not efficiently predict the prognosis or persistence of ASCUS or LSIL. The need to develop a predictive biomarker for ASCUS and LSIL progression in cervical cytology to provide more predictive information and reduce discomfort still remains.

HPV L1, one of the HPV-induced proteins, is a capsid protein produced at the upper layer of the differentiated cervical epithelium at the end of the viral life cycle. HPV is released from cervical keratinocytes after the assembly of viral proteins^8^, ^9^. HPV L1 immunocytochemistry in cervical cytology was found to be a useful predictor of high grade CIN among patients with ASCUS or LSIL^10^, ^11^. Therefore, we wanted to combine HPV L1 immunocytochemistry with the cervical cancer screening triage, which are both used individually in clinical practice, to evaluate the risk of CIN2+ in women with persistent ASCUS or LSIL.

The aim of this study was to find the best predictive model for development of CIN 2+ using the immunocytochemical expression of the HPV L1 capsid protein in patients with persistent ASCUS, and LSIL with high risk of HPV infection.

## Material and Methods

### Study subjects

The medical records of patients who visited the department of obstetrics and gynecology of Seoul St. Mary's Hospital between January 2014 and August 2017 were reviewed. Persistent LSIL or ASCUS was identified by: 1) diagnosis of ASCUS or LSIL on follow up cytology after 1 year, and 2) identification in at least two consecutive cytologic examinations. The inclusion criteria were as follows: 1) persistent ASCUS or LSIL, 2) high risk of HPV infection, and 3) presence of cervical histopathology. The exclusion criteria were as follows: 1) patients who showed high grade squamous intraepithelial lesion (HSIL) or atypical squamous cells with cannot exclude HSIL (ASC-H) at first cytologic examination, 2) low risk of HPV infection, 3) patients who were diagnosed with CIN 2 or higher for the first time, and 4) history of cervical operation or hysterectomy before the study period.

A total of 285 cervical cytology samples (70 ASCUS, and 215 LSIL Pap smears) were analyzed in this study. Cervical cytology samples were scraped from the exocervix and endocervix with a cervical brush. Cytological diagnosis was made following 2001 Bethesda system. HPV genotype was determined using the HPV DNA chip (PANArray^TM^ HPV Genotyping Chip; Pangene CO., Seoul, Korea). The cervical tissue diagnosis was obtained from the records of patients who underwent colposcopy guided biopsy, loop electrosurgical excision procedure (LEEP), or total hysterectomy. Information on age, cervical cytology, HPV genotype, expression status of HPV L1 analyzed by immunocytochemistry, and pathologic diagnosis of the cervix were collected for all patients.

The study design, data collection, and analyses followed the principles of the declaration of Helsinki. This study was approved by the Institutional Review Board (IRB) of The Catholic University of Korea (KC16RISI0388).

### Immunocytochemistry

Immunocytochemical identification of the HPV L1 capsid protein in cervical cytology samples was performed using the Cytoactive^®^ HPV L1 screening set (Cytoimmun Diagnostics GmbH, Primasens, Germany) according to the manufacturer's protocol. The coverslip and mounting medium of Papanicolaou-stained cervical smear slides in xylene were separated, and the residual xylene on the slides was removed with alcohol. To retrieve the antigen, 0.01mol/L citrate buffer (pH 6.0) was used, and the sample was heated in a microwave vacuum histoprocessor (RHS-1; Milestone, Bergamo, Italy) at a temperature of 121°C for 15 minutes. Slides were kept at room temperature for 10 minutes to cool. Subsequently, one droplet of HPV L1 capsid antibody was applied to the cervical smear slides, and slides were incubated for 1 hour at room temperature. One drop of ready -to- use- chromogen solution (3-amino-9-ethylcarbazole) was applied to the slide for 5 minutes. Mayer's hematoxylin counterstain was then applied. At least 1 positive control slide supplied by the manufacturer was included in each batch. Immunocytochemistry was performed using the first specimen.

### Statistical analysis

The expression level of the HPV L1 capsid protein in cervical cytology samples was calculated, and the correlation between HPV L1 expression and cervical pathologic diagnosis was evaluated. HPV L1 expression and other variables used to predict CIN2+ were analyzed using Fisher's exact test, χ^2^, and analysis of model comparison. All analyses were performed using SAS version 8.0 (SAS institute Inc., Cary, NC, USA). *P* <0.05 was considered statistically significant. Akaike information criterion (AIC) and Schwarz criterion (SC) were used to select of the best model to predict CIN 2+.

## Results

The sample population for this study included information from 70 patients with ASCUS and 215 patients with LSIL. The general characteristics of the study subjects are shown in Table 1. Among the patients with persistent ASCUS and LSIL, 244 (85.6%) were diagnosed with CIN1 or cervicitis and 41 (14.4%) were diagnosed with CIN2+ based on cervical histopathology.

Table 2 shows the association between the level of HPV L1 expression and cervical pathology in ASCUS and LSIL. Negative results for HPV L1 immunocytochemistry were more frequently observed in CIN2+, and positive results for HPV L1 capsid protein immunocytochemistry were more frequent in CIN 1 or cervicitis in both ASCUS and LSIL groups (Fisher's exact test, *p*<0.05). Further risk of developing CIN2+ was increased five folds in patients with ASCUS compared to that in patients showing LSIL when the HPV L1 expression was absent (Odds ratio (OR) 5.2, 95% confidence interval (CI) 2.2-12.0, *p*<0.001) (Table 3). Table 4 shows the association between the expression of the HPV L1 capsid protein and cervical pathology in patients infected with HPV types 16 ,18, and other types. In patients infected with HPV type 16 or 18, frequency of negative HPV L1 capsid protein expression was significantly increased compared to HPV L1 positive CIN2+ (*p*<0.001). In patients with other HPV types, HPV L1 negativity was more frequently observed compared to HPV L1 positivity; however, these differences were not significant (*p*=0.182). Based on previous results, HPV 16 and 18 infection and lack of HPV L1 protein expression correlated with the development of CIN 2+.

Table 5 demonstrates that the risk of developing CIN2+ was significantly higher in patients who lacked HPV L1 expression and HPV 16 or 18 infections compared to any other combination in women with both ASCUS and LSIL (OR 6.9, 95% CI 2.2-22.2, *p*=0.001, OR 6.7, 95% CI 2.8-18.3, *p*< 0.001, respectively).

Finally, the diagnostic value of various combinations of cervical cancer screening tools to predict CIN2+ was calculated (Table 6). A lower value of AIC and SC was identified as having a higher diagnostic value. Diagnosis of CIN2+ was the highest when the combination of HPV L1 capsid protein immunocytochemistry, cytology and HPV test were used when compared to other combinations (AIC: 191.7, SC: 206.3, *p*<0.001).

## Discussion

ASCUS and LSIL are generally considered to be compatible with CIN1. Therefore, a wait and see approach is generally preferred for the management of low grade cervical diseases. However, 4% to 36% of women with persistent ASCUS/LSIL as shown by cervical cytology exhibited high grade histopathology^12^, ^13^. A key finding of this study is that a combination of negative results for HPV L1 immunocytochemistry and HPV 16 or 18 infection could effectively predict development of CIN2+ in patients with persistent ASCUS and LSIL.

The present study found that HPV L1 negative cytology samples correlated with the development of CIN 2+. This finding is consistent with previous reports that the HPV L1 gene expression is decreased in higher grades of CIN. Lack of HPV L1 expression in cervical cytology may be a useful predictive marker for the detection of higher grade CIN. HPV infection is categorized into 2 phases; productive and integrative. The productive phase is characterized by HPV infection of the basal layer through micro-wounds in the epithelium, amplification of HPV viral genes within differentiating epithelial cells and assembly of the HPV L1 capsomere in the upper epithelium 14. Among HPV viral proteins, HPV L1 induces the most striking immune response, particularly in CD8+ T cells. This finding suggests that the HPV L1 protein can have a beneficial immunological role in the spontaneous regression of cervical disease^15^. Positive expression of the HPV L1 capsid protein may be a promising indicator of good prognosis of cervical disease. In contrast, the HPV L1 protein is not detected when HPV is integrated into the human genome. Thus, lack of HPV L1 protein may reflect reduced cellular immune response and thereby promote malignant transformation of cervical epithelial cells^14^. Immunologic evasion may be a critical aspect of high grade cervical lesions^16^, ^17^. Lack of HPV L1 protein expression significantly correlated with high grade CIN^2^. Even in a study of women under 30 years of age with mild cervical dysplasia, lack of HPV L1 expression associated with disease progression^18^, ^19^.

Moreover, HPV infection is associated with an increased risk of development of CIN2+ in patients with ASCUS and LSIL^20^. In contrast, in the absence of HPV, the cytologic behavior of ASCUS is similar to normal cervical cytology samples. The presence of HPV can be an important pathogenic determinant for the progression of ASCUS and LSIL. High levels of the HPV oncoproteins E6 and E7 play important roles in the progression of cervical disease. HPV E6 inactivates the tumor suppressor, p53, and E7 downregulates pRB 21. These activities enhance uncontrolled cell division in cervical disease. Compared to other high risk HPV infections, HPV 16 infection more closely correlated with the risk of CIN2+ (31.5% vs 8.6%) in the Addressing THE Need for Advanced HPV Diagnostic(ATHENA) trial^22^. Additionally, McKenna *et al* documented that HPV 16 and 18 infections highly correlated with the development of CIN3^23^. In a 10-year follow-up study, 17.2% of women with HPV 16 infection and 13.6% of women with HPV 18 showed progression to CIN3^24^. Similarly, in this study, 22.8% of the women with HPV type 16 or 18 demonstrated CIN2+ histopathology compared to 3.9% of women with other types of HPV infection. Also, lack of HPV L1 expression correlated with a significantly higher rate of CIN2+ development than positive HPV L1 expression among patients with HPV 16 or 18 infections. It is possible that the lack of HPV L1 expression is more associated with HPV 16 or 18 infections than other types of HPV infections. According to previous reports, HPV 16 is closely related to HPV integration into the host genome and the absence of HPV L1 expression as determined by immunohistochemistry^25^. Results of this study indicate that, when both HPV infection and absent HPV L1 expression are observed, patients with persistent ASCUS progressed to CIN2+ more frequently than patients with persistent LSIL. A similar trend was seen in an observational study in which women with both HPV infection and persistent ASCUS were at higher risk than women with persistent LSIL for the development of CIN3+ (6.8% vs 5.2%, *p*< 0.001) and cancer (0.4% vs 0.16%, *p*=0.04) between the ages 30 to 64^20^. Additionally, the combination of absent HPV L1 expression and HPV integration could significantly predict progression to CIN2+ among patients with ASCUS and LSIL. For this reason, colposcopic examination was recommended by the ASCCP for women 30 years of age or older with HPV 16 or 18 infection regardless of cytologic pathology^26^.

Observational data for women with persistent ASCUS and LSIL demonstrated that the risk of progression to CIN 2+ increases 1~2% annually^20^. Based on our experience in the gynecologic clinic, patients who were initially diagnosed with minor abnormal cervical cytology, they were compliant with the doctor's recommendations with a mild degree of fear. On the contrary, patients who presented with persistent ASCUS and LSIL typically expressed deep concern regarding the risk of progression to a high grade disease^27^.

Despite the high rate of spontaneous regression of ASCUS/LSIL, patients may experience substantial apprehension about the oncogenic potential of HPV infection, risks associated with low grade cervical abnormality, abnormal colposcopic findings, and additional invasive procedures. Observational management of patients with ASCUS/LSIL can be felt differently among doctors and patients. In general, gynecologists regard observation as way to avoid unnecessary treatment of low grade cervical abnormalities. Patients tend to worry about the possibility that delayed treatment may accelerate the progression to cervical cancers. If cervical biomarkers can offer additional confirmative information on the prognosis of persistent ASCUS and LSIL, the patient's levels of apprehension may be reduced.

The present study investigated the capability of immunocytochemistry on cervical cytology samples for the early prediction of high grade cervical disease among women with persistent ASCUS and LSIL. The HPV L1 expression level may provide information on whether observation or treatment is more appropriate for patients with persistent cervical abnormalities. The present results show that lack of HPV L1 expression and presence of HPV type 16 or 18 infection can accurately predict progression to CIN2+ in women showing persistent ASCUS and LSIL. In conclusion, HPV L1 immunocytochemistry and the HPV type test can predict CIN2+ in women with persistent ASCUS or LSIL.

## Figures and Tables

**Table 1:** General characteristics of study subjects (N=285)

	*N (*%)
Mean age(yrs) (range)	40.7 (20-78)
Parity	
0	75 (26.3)
1	88 (30.9)
2	92 (32.3)
≥3	30 (10.5)
Cervical cytology	
ASCUS	70 (24.6)
LSIL	215 (75.4)
Results of HPV L1 immunocytochemistry	
Negative	134 (47.0)
Positive	151 (53.0)
HPV Type	
16 or 18	158 (55.4)
other	127 (44.6)
Pathologic diagnosis methods	
Cervical biopsy	161 (48.2)
LEEP / Conization	144 (43.1)
Hysterectomy	29 (8.7)
Results of cervical pathology	
<CIN 2^†^	244 (85.6)
≥CIN 2^‡^	41 (14.4)

ASCUS: Atypical squamous cells of unknown significance, LSIL: Low grade squamous intraepithelial lesion, LEEP: Loop electrosurgical excision procedure, CIN: cervical intraepithelial neoplasia.^†^ It includes chronic cervicitis, CIN1, koilocytosis, and negative.^‡^ It includes CIN2, CIN3 , CIS, AIS and cervical cancer.

**Table 2:** Association between HPV L1 immunocytochemistry and cervical histopathology according to cervical cytology (N=285)

Cytology	L1 capsid protein immunocytochemistry	Pathology		*P*-value
		< CIN 2 (n=244)	≥CIN 2 (n=41)	
ASCUS (n=70)	L1 negative	22(46.8)	19(82.6)	0.005^†^
	L1 positive	25(53.2)	4(17.4)	
LSIL (n=215)	L1 negative	80(40.6)	13(72.2)	0.01^†^
	L1 positive	117(59.4)	5(27.8)	

**Table 3:** Comparison of risk of development of CIN2+ between ASCUS and LSIL according to HPV L1 protein status (N=285)

CIN2+		
L1 capsid protein immunochemistry		
Cytology	L1 negativity	L1 positivity
ASCUS	5.2(2.2-12.0)†	3.8(0.9-14.3)†
LSIL(ref)	1	1
P- value	<0.001	0.051

^†^ Odds ratio (95% confidence interval)

**Table 4:** Association between HPV L1 immunocytochemistry and cervical histopathology according to HPV types (N=285)

HPV infection type	HPV L1 capsid protein immunocytochemistry	Pathology < CIN 2 ≥ CIN2 (n=244) (n=41)		*P*-value
16 or 18	L1 negative (n=75)	47(38.5)	28(77.8)	<0.001^*^
	L1 positive (n=83)	75(61.5)	8(22.2)	
Other types	L1 negative (n=59)	55(45.1)	4(80.0)	0.182^†^
	L1 positive (n=68)	67(54.9)	1(20.0)	

^*^Chi-square test, ^†^Fisher's exact test

**Table 5:** Association between HPV immunocytochemistry with HPV types and cervical histopathology according to cervical cytology (N=285)

Cytology	HPV infection status and HPV L1 capsid protein immunocytochemistry	<CIN 2 (n=244)	≥CIN 2 (n=41)	OR (95% CI)	P-value
ASCUS	HPV 16 or 18 infection andHPV L1 protein negative (n=34)	16 (34.1)	18 (78.3)	6.9 (2.2, 22.2)	0.001
	Others(n=36)†	31 (65.9)†	5 (21.7)†	1 (Ref)†	
LSIL	HPV 16 or18 infection and HPV L1protein negative(n=41)	31 (15.7)	10 (55.6)	6.7 (2.5, 18.3)	<0.001
	Others (n=174)†	166 (84.3)†	8 (44.4)†	1 (Ref)†	

^†^ HPV 16 or 18 or other type infection with HPV L1 protein negative or positive

**Table 6:** Diagnostic value to predict CIN2+ using combinations of cervical cancer screening tools (N=285)^*^

									Backward	Forward
Model	Cytology		Cytology		HPV test		AIC^†^	SC^‡^	*P-*value	*P*-value
	ASCUS (n=70)	LSIL (n=215)	Positive (n=151)	Negative (n=134)	16 or 18 (n=158)	Others (n=127)				
	OR (95% CI)		OR (95% CI)		OR (95% CI)					
Cytology+L1 protein+ HPV	2.93 (1.37-6.29)	1	0.19 (0.1-0.4)	1	5.6 (1.9-15.6)	1	191.7	206.3	0.0002	<0.0001
Cytology+L1 protein	4.83 (2.35-9.91)	1	0.2 (0.1-0.5)	1			203.1	214.0	<0.0001	<0.0001
Cytology+ HPV	3.3 (1.58-6.88)	1			4.7 (1.7-12.9)	1	207.5	218.4	<0.0001	<0.0001
Cytology	5.36 (2.68-10.72)	1					216.4	223.7	<0.0001	<0.0001
Intercept only							236.8	234.8	<0.0001	<0.0001

^*^Model comparison, ^†^Akaike information criterion, ^‡^Schwarz criterion
